# Emotional responses of Korean and Chinese women to Hangul phonemes to the gender of an artificial intelligence voice

**DOI:** 10.3389/fpsyg.2024.1357975

**Published:** 2024-07-29

**Authors:** Min-Sun Lee, Gi-Eun Lee, San Ho Lee, Jang-Han Lee

**Affiliations:** ^1^Department of Psychology, Chung-Ang University, Seoul, Republic of Korea; ^2^Institute of Cultural Diversity Content, Chung-Ang University, Seoul, Republic of Korea; ^3^Department of European Language and Cultures, Chung-Ang University, Seoul, Republic of Korea

**Keywords:** phoneme, arousal, valence, emotions, sound symbolism, artificial intelligence voice

## Abstract

**Introduction:**

This study aimed to explore the arousal and valence that people experience in response to Hangul phonemes based on the gender of an AI speaker through comparison with Korean and Chinese cultures.

**Methods:**

To achieve this, 42 Hangul phonemes were used, in a combination of three Korean vowels and 14 Korean consonants, to explore cultural differences in arousal, valence, and the six foundational emotions based on the gender of an AI speaker. A total 136 Korean and Chinese women were recruited and randomly assigned to one of two conditions based on voice gender (man or woman).

**Results and discussion:**

This study revealed significant differences in arousal levels between Korean and Chinese women when exposed to male voices. Specifically, Chinese women exhibited clear differences in emotional perceptions of male and female voices in response to voiced consonants. These results confirm that arousal and valence may differ with articulation types and vowels due to cultural differences and that voice gender can affect perceived emotions. This principle can be used as evidence for sound symbolism and has practical implications for voice gender and branding in AI applications.

## Introduction

Artificial intelligence (AI), which can be considered the core technology of the Fourth Industrial Revolution, has various applications. In particular, speakers, an important function of AI, facilitate communication between humans and AI. However, a challenge remains whether AI speakers can evolve from providing basic convenience functions, such as weather notifications and alarm settings, to providing better human–machine interaction. Previous studies on emotion recognition and emotional expression of AI have been actively conducted for better comprehensive communication. Most studies aimed to determine whether AI could recognize and express human-like emotions based on a user's emotional state. However, communication is two-way in nature. Thus, it is important to consider not only the emotions conveyed by AI but also how humans perceive the messages delivered by AI speakers. In the context of marketing channels, when users of AI speakers experience positive rather than negative emotions with AI speakers, their preference for these devices increases (Jang and Ju, 2019).

Humans communicate and express and spread emotions through language. Notably, the rapid identification of emotional elements in sound stimuli of communication plays an important role in survival and adaptation (Ramachandran and Hubbard, 2001). According to sound symbolism, specific phonemes, which are the fundamental elements of sound in a language, convey meaning independently (Lowrey et al., 2003). The *Bouba–Kiki effect*, a good example of sound symbolism, refers to the phenomenon of how people associate round shapes when they hear “Bouba” and pointed shapes when they hear “Kiki” (Ramachandran and Hubbard, 2001; Maurer et al., 2006; Pejovic and Molnar, 2017). Similarly, the *gleam–glum effect* demonstrates that words containing /i;/, such as “gleam,” are perceived as more positive emotions than words containing /Λ/, such as “glum” (Yu, 2021). However, little agreement exists on whether these effects are universally applicable, regardless of the native language or age.

The question of whether phonemes have sound symbolism remains unanswered (Slunecko and Hengl, 2006). Although some studies have indicated a common theme of sound symbolism, the results vary, which is likely because the phonemes can be classified into consonants and vowels. Recent studies have revealed that the Bouba–Kiki effect varies between Eastern and Western cultures (Chen et al., 2016) and can change depending on differences in native language (Styles and Gawne, 2017). These previous findings suggest that sound–shape mapping related to consonants may be influenced by individual perceptual style and linguistic experience (Rogers and Ross, 1975; Chen et al., 2016; Shang and Styles, 2017; Chang et al., 2021).

This study adopted the classification of consonants as plain, aspirated, and voiced consonants, which is a common method and is recognized to evoke similar emotional impressions in various languages, including English and Korean. However, sound–size mapping associated with vowels is a common phenomenon across cultures and languages because of its lack of sensitivity to cultural backgrounds or native languages (Shinohara and Kawahara, 2010; Hoshi et al., 2019; Chang et al., 2021).

Therefore, vowels were selected based on the symbolism of vowel sounds. For instance, in the early research on sound symbolism by Sapir (1929), experiments were conducted on the size symbolism of vowels /a/ and /i/ using meaningless words “mal” and “mil.” Participants were asked to identify those words that referred to a large table and a small table. Approximately 80% of participants indicated that “mal” denoted a large table and “mil” denoted a small table. This suggests that /a/, when added to an existing word, conveys a soft feeling because it is a central and low vowel, indicating augmentation for distant or large objects or long durations. Conversely, /i/ is considered to represent close and small objects or short durations. These research findings highlight the influence of mouth shape during pronunciation. In terms of the dimension of the aperture, high vowels, such as /i/ or /u/, involve a smaller aperture, whereas low vowels, such as /a/, involve a larger aperture, potentially conveying different symbolic meanings (Shinohara and Kawahara, 2010). Based on this evidence, this study adopted three representative vowel types that can induce different states and constructed 42 combinations of consonants and vowels.

To date, research on sound symbolism has mainly focused on vowels rather than consonants because consonants cannot be pronounced without vowels; thus, the sound symbols of vowels have been considered greater than those of consonants (Aveyard, 2012). However, actual language cannot ignore the influence of consonants, and comparing only the differences in vowels can limit recognition of the emotional meaning. Therefore, considering the practicality of language, this study attempts to measure the emotional values of both vowels and consonants through classification according to the articulation method (Kim, 2019).

When evaluating the emotional values of stimuli, arousal and valence are the two most basic dimensions (Russell, 1980). Arousal is evaluated based on how exciting or arousing a stimulus is, that is, how calm it is, and valence is evaluated based on how pleasant or unpleasant a stimulus is. According to empirical studies, the arousal and valence dimensions are not independent of each other and exhibit a *U*-shaped relationship. Thus, unpleasant stimuli are considered more arousing than pleasant stimuli, and both unpleasant and pleasant stimuli are more arousing than neutral stimuli (Libkuman et al., 2007; Grühn and Scheibe, 2008). In general, negative emotional stimuli are considered to have a higher arousal value than positive or neutral stimuli (Ekman et al., 1983).

However, preferences for words that express emotions show differences, indicating that cultural differences can also occur between language and emotional meanings (Park et al., 2018). Thus, even in the same situation that evokes emotions, the terms cognitively interpreted and referred to differ across cultures, and depending on how emotional words are translated, they can have distinct meanings (Hahn and Kang, 2000). Furthermore, many studies have measured the properties of sounds, such as rough, soft, strong, or weak. However, because of the ambiguous nature of these adjectives and their lack of integration into each study, accurately classifying how people feel about sound is not possible. Considering these points, this study adopted universal emotions to distinguish between subjective emotional states. The six basic emotions, namely anger, disgust, fear, sadness, surprise, and happiness (Ekman and Oster, 1979), were used to measure subjective emotional states instead of relying on somewhat ambiguous emotional expressions (e.g., softness, strength, weakness, and sharpness) based on the degree of arousal and valence (Russell, 1980, 2003; Barrett, 2006a,b).

The effect of AI speakers on human emotion recognition may include variables such as the voice gender of AI speakers, as well as sound-shape and sound-size. Studies have demonstrated differences in preference for “themes” by voice gender (Kim and Yun, 2021) and in AI usage behavior based on human gender and experience (Ji et al., 2019; Obinali, 2019; Ernst and Herm-Stapelberg, 2020; Kim and Yun, 2021; Wang et al., 2021). For example, in “warm news” delivery, female voices are highly appreciated in terms of understanding, reliability, and favorability, whereas, for news with serious content, male voices are preferred (Kim and Yun, 2021). Thus, gender preferences for an AI speaker may differ according to the gender of the human listener. Although arousal and valence can be perceived in phoneme units, studies on the voice gender of AI speakers have not yet observed an effect of voice gender on phoneme units.

Recently, active research has been conducted on how emotions are expressed and recognized in literary works using AI-based natural language processing and machine learning techniques. In addition, studies and reflections on AI-generated speech and listeners' emotional responses have rapidly evolved in recent years (Val-Calvo et al., 2020). Particularly, with advancements in speech recognition technology, there is growing interest in exploring how the tone and expression used by AI when speaking can evoke emotional responses in listeners (Poon-Feng et al., 2014; Zheng et al., 2015). Such studies provide crucial insights into understanding the impact of AI speech technology on people's emotional responses, aiming to offer important insights for the effective development and application of this technology.

This study used a cultural comparison to explore the arousal and valence that people experience in response to Hangul phonemes according to the gender of an AI speaker. For this purpose, the most basic unit, the Hangul phoneme, was used as an experimental stimulus to evaluate arousal and valence in Korean and Chinese women who can speak Korean. This study aimed to examine the cultural differences in arousal and valence using the articulation method, vowels, and the gender of the AI speaker.

## Materials and methods

### Participants and procedure

Using G^*^Power 3.1.9.7 (the University of Düsseldorf, Düsseldorf, Germany), a power analysis was conducted with an effect size of 0.25, an alpha error probability of 0.05, a power of 0.80, and the number of groups set to 4. The analysis showed that the minimum sample size required was 128 participants (42 participants per condition). In total, 136 participants were recruited (136 women; *M*_age_ = 27.19 years, *SD* = 4.30) from a university bulletin board in South Korea. The sample consisted of 68 Korean and Chinese participants who were randomly assigned to one of two conditions in a between-subjects design with voice gender (man or woman). All participants were informed that they had been recruited for a psychological experiment measuring emotions for phonemes and that all experimental processes would be conducted online. The study was limited to women to present the differences in variables, considering that women generally exhibit greater emotional responsiveness than men. The inclusion criteria for selecting participants were as follows: women who were (1) over the age of 20 years, (2) of Korean or Chinese nationality, and (3) able to speak Korean. Before taking part in the experiment, all participants provided informed consent and were informed that they could stop the experiment at any time. Each participant received $20 for their participation.

### Measures

To control for emotional variables, the participants were asked to complete a questionnaire, described below. No significant differences were found in psychological characteristics between groups (Table 1).

**Table 1:** Psychometric characteristics of the participants.

**Measure**	**Korean (*n* = 68)**	**Chinese (*n* = 68)**	***t* (*P*-value)**
PANAS-P	27.32 ± 5.26	28.56 ± 6.23	−1.25 (0.21)
PANAS-N	25.07 ± 5.69	26.97 ± 7.75	−1.63 (0.11)
STAI-T	47.29 ± 10.20	46.82 ± 6.77	0.32 (0.75)
STAI-S	46.68 ± 10.41	46.57 ± 6.93	0.068 (0.95)
CES-D	20.01 ± 10.83	18.15 ± 9.62	1.06 (0.29)

*N* = 136; all correlations are considered not statistically significant at *P* < 0.01. Mean ± standard deviation; PANAS-P, Positive and Negative Affect Schedule Scale-Positive; PANAS-N, Positive and Negative Affect Schedule Scale-Negative; STAI-T, State-Trait Anxiety Inventory-Trait Anxiety; STAI-S, State-Trait Anxiety Inventory-State Anxiety; CES-D, Center for Epidemiologic Studies Depression Scale.

#### Positive and Negative Affect Schedule Scale

The Korean version (K-PANAS; Lee et al., 2003) and the Chinese version (C-PANAS; Huang et al., 2003) of the PANAS were used to evaluate the positive and negative affects. The PANAS comprises 20 items, with 10 evaluating expectations for positive affect (PANAS-P) and 10 evaluating expectations for negative affect (PANAS-N). Participants were asked to rate their responses on a 5-point Likert scale, where 1 indicates “very slightly or not at all” and 5 indicates “extremely.” The higher the score, the higher the levels of positive and negative affect. Cronbach's alpha values were 0.60 and 0.83 for the K-PANAS-P and C-PANAS-P, respectively, and 0.61 and 0.85 for the K-PANAS-N and C-PANAS-N, respectively.

#### State-Trait Anxiety Inventory

The Korean version (K-STAI; Kim and Shin, 1978) and the Chinese version (C-STAl; Tsoi et al., 1986) of the STAI were used to measure state and trait anxiety (Spielberger et al., 1970). This scale consists of 40 items, with 20 measuring “trait anxiety (STAI-T),” and 20 measuring “state anxiety (STAI-S).” Participants were asked to rate on a 4-point Likert scale, where 1 indicates “not at all” and 4 indicates “very much so.” The higher the score, the more intense or more often an individual felt anxious. Cronbach's alpha values of the K-STAI-T and K-STAI-S were 0.91 and 0.92, respectively, and those of the C-STAI-T and C-STAI-S were 0.75 and 0.74, respectively.

#### Center for Epidemiologic Studies Depression Scale

The Korean version (K-CES-D; Chon et al., 2001) and the Chinese version (C-CES-D; Chi and Boey, 1993) of the CES-D were used to measure the baseline for depressive mood in participants (Radloff, 1977). This scale consists of 20 items, and participants answer how often each item had occurred over the past week, with four response options ranging from 0, indicating “rarely,” to 3, indicating “all times.” Cronbach's alpha values of the K-CES-D and C-CES-D were 0.93 and 0.90, respectively.

### Stimuli

For the experiment, 42 Hangul phonemic stimuli with artificial human sounds were created and used with a text-to-speech (TTS) program. All stimuli had an equal duration of 500 ms. Considering participants' fatigue, 42 voice stimuli were used combining three Korean vowels and 14 Korean consonants (Table 2). The three vowels used were those with the largest differences in pronunciation structure (Lee and Lee, 2000), and the 14 consonants used excluded double consonants. These consonants were classified into three types according to the articulation system of classification (Kim, 2019). Specifically, they were classified as lenis consonants if they did not require heavy breathing or straining of the throat, aspirated consonants if they involved the release of a burst of strong air during plosive sounds, and voiced consonants if they resonated in the mouth or nose when pronounced.

**Table 2:** Forty-two Hangul phonetic values.

**Articulation**	**Code**	**Corner vowels**		

		ㅏ**/a/**	ㅜ**/u/**	ㅣ**/i/**
Lenis	ㄱ /g, k/	가 /ga, ka/	구 /gu, ku/	기 /gi, ki/
	ㄷ /d, t/	다 /da, ta/	두 /du, tu/	디 /di, ti/
	ㅂ /b, p/	바 /ba, pa/	부 /bu, pu/	비 /bi, pi/
	ㅅ /s/	사 /sa/	수 /su/	시 /si/
	ㅈ /j/	자 /ja/	주 /ju/	지 /ji/
	ㅎ /h/	하 /ha/	후 /hu/	히 /hi/
Aspirated	ㅊ /ch/	차 /cha/	추 /chu/	치 /chi/
	ㅋ /k/	카 /ka/	쿠 /ku/	키 /ki/
	ㅌ /t/	타 /ta/	투 /tu/	티 /ti/
	ㅍ /p/	파 /pa/	푸 /pu/	피 /pi/
Voiced	ㄴ /n/	나 /na/	누 /nu/	니 /ni/
	ㄹ /l, r/	라 /la, ra/	루 /lu, ru/	리 /li, ri/
	ㅁ /m/	마 /ma/	무 /mu/	미 /mi/
	ㅇ /ng/	아 /a/	우 /u/	이 /i/

English Notation for Articulation in Korean.

### Data analysis

A dataset with 136 samples was included in the final analysis. As the first step in the analysis, a 2 (nationality: Korean, Chinese) × 3 (articulation: lenis, aspirated, voiced), 2 (nationality: Korean, Chinese) × 3 (vowel: /a/, /u/, /i/) two-way analysis of variance (ANOVA) was conducted to identify differences in sound symbolism between participants of different nationalities. In addition, a 2 (nationality: Korean, Chinese) × 2 (voice gender: female, male) two-way ANOVA was conducted to explore the arousal and valence patterns according to nationality and voice gender. A 2 (voice gender: female, male) × 3 (articulation: lenis, aspirated, voiced) and 2 (voice gender: female, male) × 3 (vowel: /a/, /u/, /i/) mixed ANOVA was conducted with Korean and Chinese participants, respectively, to explore the patterns of arousal and valence for each nationality. An independent sample *t*-test was performed for continuous variables to compare psychological characteristics and perform planned comparisons.

## Results

### Differences between nationalities for articulation and vowels

Arousal, valence, and basic emotions were used as dependent variables, three types of articulation (lenis, aspirated, and voiced) and three vowels (/a/, /u/, and /i/) were used as within-subjects factors and nationality (Korean or Chinese) was used as a between-subjects factor to perform the repeated-measures ANOVA.

**Figure 1:**
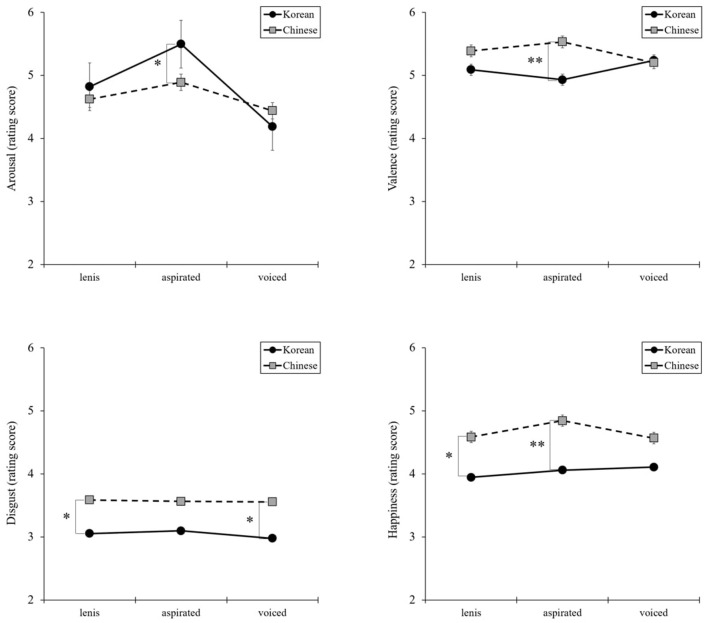
Interaction effects between the three types of articulation and nationality in arousal, valence, and basic emotions: disgust and happiness. *P < 0.05, **P < 0.01.

The results presented in Figure 1 indicate that the interaction between the three types of articulation and nationality was significant for arousal [*F*_(2, 268)_ = 11.93, *P* = 0.00, η^2^ = 0.08] and valence [*F*_(2, 268)_ = 7.33, *P* = 0.001, η^2^ = 0.05]. Then, planned comparisons were performed using independent sample *t*-tests, which revealed no differences between Korean and Chinese participants for lenis or voiced articulation. However, for aspirated articulation, arousal was higher in Chinese participants than in Korean participants [*t*_(134)_ = 2.51, *P* = 0.01, *d* = 0.43], whereas valence was higher in Korean than Chinese participants [*t*_(134)_ = −3.09, *P* = 0.00, *d* = 0.53]. Furthermore, significant interactions were observed between two basic emotions. At the lenis and voiced articulation levels, disgust was higher in Chinese participants than in Korean participants [*t*_(134)_ = −1.99, *P* = 0.49, *d* = 0.34; *t*_(134)_ = −1.985, *P* = 0.049, *d* = 0.34), and at the lenis and aspirated articulation levels, happiness was higher in Chinese participants than in Korean participants [*t*_(134)_ = −2.61, *P* = 0.01, *d* = 0.45; *t*_(134)_ = −2.93, *P* = 0.004, *d* = 0.50].

**Figure 2:**
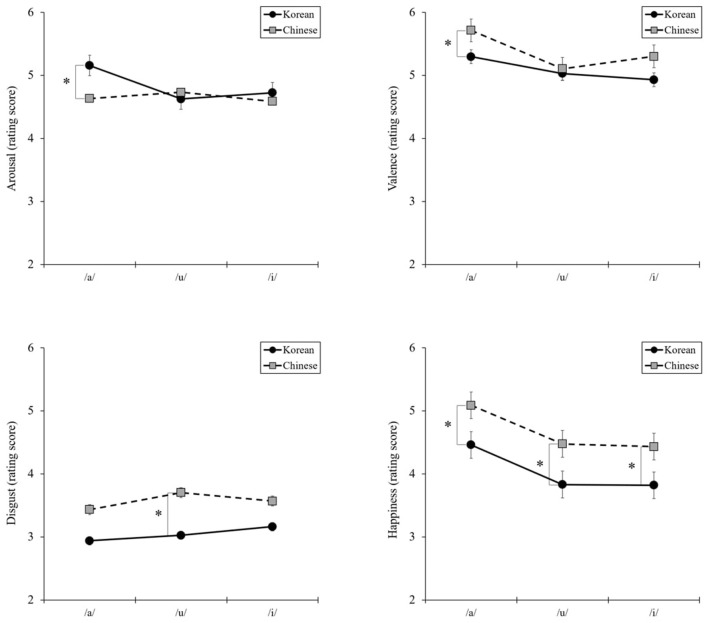
Interaction effects between three vowels and nationality in arousal, valence, and basic emotions: disgust and happiness. *P < 0.05.

Similar results were found for vowels. As shown in Figure 2, the interaction between three vowels and nationality was significant in arousal [*F*_(2, 268)_ = 8.73, *P* = 0.000, η^2^ = 0.06] and valence [*F*_(2, 268)_ = 3.31, *P* = 0.04, η^2^ = 0.02]. Planned comparisons performed using independent sample *t*-tests showing no differences were found between Korean and Chinese for /u/ or /i/. However, for /a/, arousal was higher in Korean participants than in Chinese participants [*t*_(134)_ = 2.40, *P* = 0.02, *d* = 0.41], whereas the valence was higher in Chinese participants than in Korean participants [*t*_(134)_ = −2.08, *P* = 0.04, *d* = 0.36]. Furthermore, significant interactions were observed between the two basic emotions. For /u/, disgust was higher in Chinese participants than in Korean participants [*t*_(134)_ = −2.40, *P* = 0.02, *d* = 0.41], and for all vowels, happiness was higher in Chinese participants than in Korean participants [/a/: *t*_(134)_ = −2.32, *P* = 0.02, *d* = 0.40; /u/: *t*_(134)_ = −2.59, *P* = 0.01, *d* = 0.44; /i/: *t*_(134)_ = −2.35, *P* = 0.02, *d* = 0.40]. However, no significant interaction effects were found for the other basic emotions.

### Differences between nationalities for articulation and vowels

**Figure 3:**
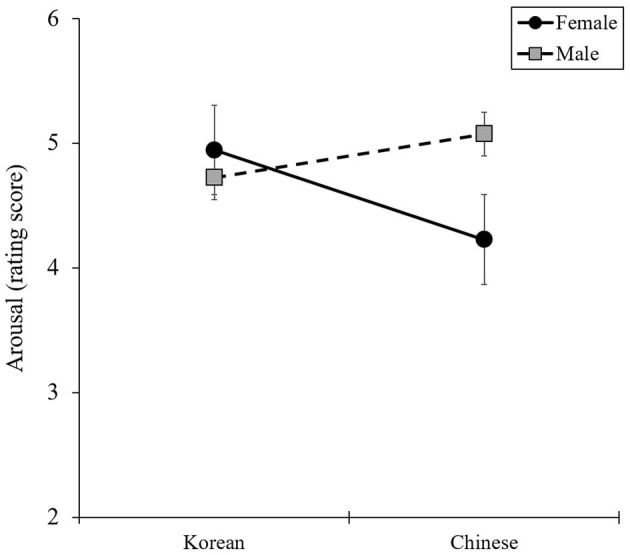
Interaction effects between nationality and voice gender in arousal.

The results of the two-way ANOVA, presented in Figure 3, provide statistical support for the interaction between nationality (Korean or Chinese) and voice gender (female or male) for arousal [*F*_(1, 132)_ = 7.92, *P* = 0.006, η^2^ = 0.06]. However, no significant interaction effects were found for valence or basic emotions.

**Figure 4:**
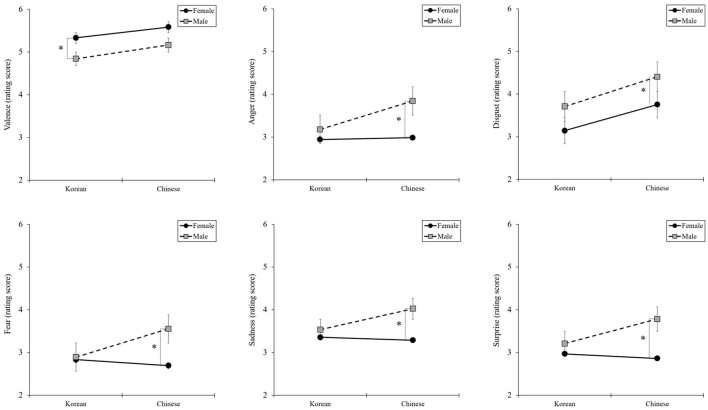
Simple main effect analysis of nationality and voice gender. *P < 0.05.

No interaction effects were found on the valence or basic emotional score. However, the one-way ANOVA for simple main-effect analysis revealed differences in voice gender by nationality (Figure 4). Unlike Korean participants, Chinese participants reported feeling more negatively toward male voices than female voices.

### Differences in the value of articulation for voice gender by nationality

**Figure 5:**
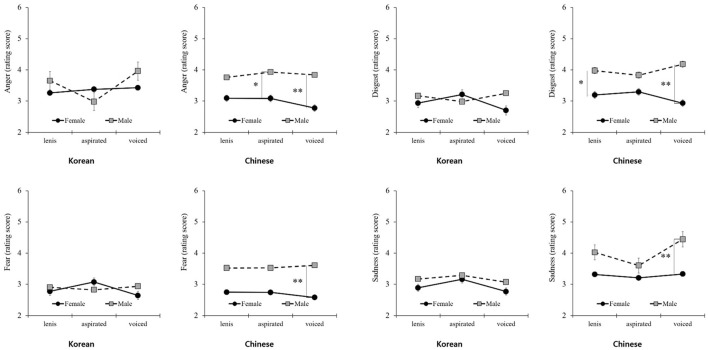
Differences in basic emotions for voice gender by nationality. *P < 0.05, **P < 0.01.

As shown in Table 3, the articulation types showed differences in arousal based on voice gender by nationality. Aspirated articulation elicited more arousal than voiced articulation, regardless of voice gender. However, unlike Korean participants, Chinese participants showed significant differences in arousal based on voice gender. In particular, for aspirated and voiced articulation, articulation ratings were significantly higher for a male voice than for a female voice. However, valence exhibited a different pattern for the male voice compared to the female voice. Although arousal was rated lower for the female voice than for the male voice, valence was rated higher for the female voice than for the male voice. Regarding basic emotions, clear differences were observed in the patterns between nationalities (Figure 5). Korean participants showed no significant differences in scores regardless of voice gender, whereas Chinese participants exhibited a difference in scores between voice and gender, especially for voiced articulation.

**Table 3:** Differences in the types of articulation for voice gender by nationality.

		**Nationality**	**Voice gender**		***F***

			**Female**	**Male**	
Arousal	Lenis	Korean	4.94 ± 0.66	4.71 ± 1.04	1.19
		Chinese	4.31 ± 1.56	4.94 ± 1.03	3.87
	Aspirated	Korean	5.68 ± 1.15	5.31 ± 1.40	1.38
		Chinese	4.52 ± 1.80	5.27 ± 1.08	4.31^*^
	Voiced	Korean	4.23 ± 1.01	4.15 ± 1.27	0.07
		Chinese	3.86 ± 1.57	5.03 ± 1.05	13.00^**^
Valence	Lenis	Korean	5.38 ± 0.67	4.81 ± 1.04	7.27^**^
		Chinese	5.54 ± 1.57	5.23 ± 0.72	1.11
	Aspirated	Korean	4.76 ± 0.92	5.14 ± 1.08	2.83
		Chinese	5.61 ± 1.52	5.45 ± 0.89	0.268
	Voiced	Korean	5.89 ± 1.06	4.59 ± 1.24	21.87^***^
		Chinese	5.60 ± 1.75	4.81 ± 0.76	5.83^*^
Anger	Lenis	Korean	2.89 ± 1.44	3.17 ± 1.58	0.60
		Chinese	3.09 ± 1.58	3.76 ± 1.55	3.08
	Aspirated	Korean	3.16 ± 1.63	3.29 ± 1.69	0.10
		Chinese	3.09 ± 1.60	3.93 ± 1.69	4.48^*^
	Voiced	Korean	2.77 ± 1.68	3.07 ± 1.66	0.56
		Chinese	2.78 ± 1.54	3.84 ± 1.53	8.21^**^
Disgust	Lenis	Korean	2.94 ± 1.55	3.17 ± 1.57	0.38
		Chinese	3.20 ± 1.61	3.98 ± 1.46	4.39^*^
	Aspirated	Korean	3.21 ± 1.67	2.98 ± 1.54	0.35
		Chinese	3.30 ± 1.78	3.83 ± 1.60	1.70
	Voiced	Korean	2.70 ± 1.70	3.25 ± 1.65	1.83
		Chinese	2.93 ± 1.61	4.18 ± 1.63	10.13^**^
Fear	Lenis	Korean	2.78 ± 1.55	2.91 ± 1.60	0.12
		Chinese	2.75 ± 1.60	3.53 ± 1.61	3.96
	Aspirated	Korean	3.08 ± 1.61	2.83 ± 1.57	0.42
		Chinese	2.74 ± 1.62	3.53 ± 1.70	3.79
	Voiced	Korean	2.65 ± 1.77	2.94 ± 1.67	0.51
		Chinese	2.58 ± 1.70	3.61 ± 1.62	6.51^*^
Sadness	Lenis	Korean	3.26 ± 1.44	3.65 ± 1.69	1.06
		Chinese	3.32 ± 1.71	4.03 ± 1.41	3.47
	Aspirated	Korean	3.38 ± 1.64	2.98 ± 1.30	1.22
		Chinese	3.21 ± 1.66	3.61 ± 1.42	1.12
	Voiced	Korean	3.43 ± 1.62	3.96 ± 1.87	1.59
		Chinese	3.33 ± 1.54	4.44 ± 1.35	10.03^**^
Surprise	Lenis	Korean	2.90 ± 1.54	3.17 ± 1.60	0.50
		Chinese	2.94 ± 1.62	3.74 ± 1.51	4.49^*^
	Aspirated	Korean	3.20 ± 1.66	3.50 ± 1.78	0.52
		Chinese	2.98 ± 1.59	3.90 ± 1.56	5.72^*^
	Voiced	Korean	2.81 ± 1.67	2.96 ± 1.54	0.16
		Chinese	2.66 ± 1.49	3.71 ± 1.55	8.06^**^
Happiness	Lenis	Korean	3.93 ± 1.31	3.96 ± 1.50	0.01
		Chinese	4.60 ± 1.61	4.57 ± 1.33	0.01
	Aspirated	Korean	3.59 ± 1.41	4.53 ± 1.679	6.155^*^
		Chinese	4.71 ± 1.65	4.98 ± 1.373	0.507
	Voiced	Korean	4.44 ± 1.58	3.78 ± 1.664	2.851
		Chinese	4.91 ± 1.63	4.22 ± 1.411	3.505

^*^P < 0.05, ^**^P < 0.01, ^***^P < 0.001.

### Differences in the value of vowels for voice gender by nationality

**Figure 6:**
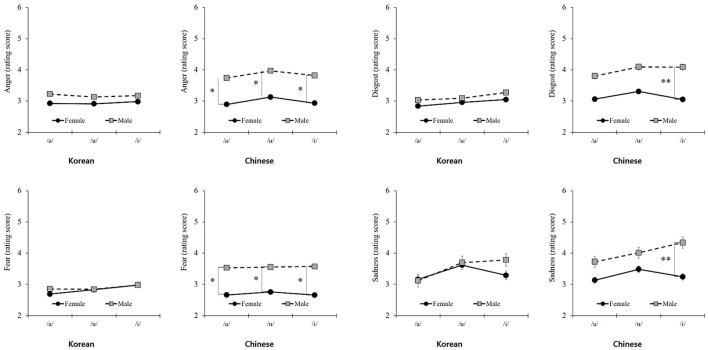
Differences in basic emotions for voice gender by nationality. *P < 0.05, **P < 0.01.

As presented in Table 4, differences were found in arousal for the three vowels based on voice gender by nationality. Unlike the articulation results, for vowels, the patterns of arousal for voice gender differed depending on nationality. In Chinese participants, the difference in arousal value based on voice gender was remarkable for all types; however, in Korean participants, a difference in arousal value based on voice gender was found only for /i/. Regarding basic emotions, clear differences were observed in the patterns between nationalities. For Korean participants, the scores were not significantly different, regardless of voice gender. However, for Chinese participants, scores differed between voice and gender (Figure 6).

**Table 4:** Differences in the value of vowels for voice gender by nationality.

		**Nationality**	**Voice gender**		***F***

			**Female**	**Male**	
Arousal	/a/	Korean	5.13 ± 0.82	5.19 ± 1.18	0.07
		Chinese	4.14 ± 1.66	5.13 ± 1.10	8.24^**^
	/u/	Korean	4.64 ± 0.72	4.61 ± 1.33	0.01
		Chinese	4.36 ± 1.66	5.10 ± 0.99	5.0^*^
	/i/	Korean	5.10 ± 0.85	4.37 ± 1.12	8.59^**^
		Chinese	4.18 ± 1.67	5.00 ± 1.00	6.13^*^
Valence	/a/	Korean	5.48 ± 0.74	5.12 ± 1.09	2.49
		Chinese	5.88 ± 1.72	5.56 ± 0.86	0.94
	/u/	Korean	5.29 ± 0.85	4.78 ± 1.14	4.35^*^
		Chinese	5.22 ± 1.52	4.99 ± 0.85	0.59
	/i/	Korean	5.23 ± 0.74	4.63 ± 1.12	6.84^*^
		Chinese	5.66 ± 1.62	4.95 ± 0.94	4.92^*^
Anger	/a/	Korean	2.93 ± 1.58	3.23 ± 1.74	0.55
		Chinese	2.90 ± 1.43	3.74 ± 1.53	5.54^*^
	/u/	Korean	2.91 ± 1.55	3.13 ± 1.70	0.31
		Chinese	3.13 ± 1.72	3.97 ± 1.57	4.45^*^
	/i/	Korean	2.98 ± 1.53	3.18 ± 1.66	0.25
		Chinese	2.93 ± 1.53	3.80 ± 1.64	5.33^*^
Disgust	/a/	Korean	2.84 ± 1.59	3.04 ± 1.64	0.25
		Chinese	3.06 ± 1.59	3.80 ± 1.66	3.53
	/u/	Korean	2.96 ± 1.65	3.09 ± 1.58	0.11
		Chinese	3.31 ± 1.83	4.10 ± 1.46	3.85
	/i/	Korean	3.05 ± 1.52	3.28 ± 1.66	0.34
		Chinese	3.05 ± 1.62	4.09 ± 1.52	7.36^**^
Fear	/a/	Korean	2.69 ± 1.64	2.86 ± 1.58	0.17
		Chinese	2.66 ± 1.64	3.53 ± 1.66	4.75^*^
	/u/	Korean	2.83 ± 1.61	2.85 ± 1.58	0.00
		Chinese	2.76 ± 1.68	3.56 ± 1.53	4.18^*^
	/i/	Korean	2.98 ± 1.63	2.98 ± 169	0.00
		Chinese	2.66 ± 1.58	3.58 ± 1.72	5.24^*^
Sadness	/a/	Korean	3.16 ± 1.56	3.12 ± 1.48	0.01
		Chinese	3.13 ± 1.60	3.72 ± 1.46	2.54
	/u/	Korean	3.62 ± 1.65	3.70 ± 1.62	0.04
		Chinese	3.48 ± 1.75	4.01 ± 1.29	2.01
	/i/	Korean	3.29 ± 1.55	3.78 ± 1.75	1.52
		Chinese	3.24 ± 1.57	4.34 ± 1.51	8.59^**^
Surprise	/a/	Korean	2.94 ± 1.61	3.58 ± 1.78	2.45
		Chinese	3.01 ± 1.47	3.83 ± 1.45	5.37^*^
	/u/	Korean	2.99 ± 1.67	3.11 ± 1.65	0.09
		Chinese	2.80 ± 1.63	3.84 ± 1.61	7.04^*^
	/i/	Korean	2.98 ± 1.54	2.94 ± 1.52	0.01
		Chinese	2.78 ± 1.57	3.68 ± 1.65	5.35^*^
Happiness	/a/	Korean	4.36 ± 1.95	4.56 ± 1.76	0.26
		Chinese	5.00 ± 1.74	5.17 ± 1.42	0.20
	/u/	Korean	3.72 ± 1.46	3.95 ± 1.46	0.43
		Chinese	4.53 ± 1.59	4.43 ± 1.32	0.07
	/i/	Korean	3.88 ± 1.34	3.76 ± 1.63	0.12
		Chinese	4.70 ± 1.64	4.17 ± 1.42	2.04

^*^P < 0.05, ^**^P < 0.01, ^***^P < 0.001.

## Discussion and conclusion

This study aimed to explore cultural differences by comparing the degree of arousal and valence experienced by Korean and Chinese women in Hangul phonemes, based on the gender of an AI voice. The results of this study revealed significant differences in arousal levels between Korean and Chinese women in response to male AI voices. In particular, Chinese women exhibited distinct differences in emotional perceptions of male and female voices in voiced consonants. In addition, this study classified participants by nationality and identified cultural differences in arousal and valence patterns according to articulation and vowels.

This study revealed that arousal and valence levels differed between Korean and Chinese women, even for phonemic units without conceptual meaning. This is consistent with Russell's claim that emotional stimuli may have cultural differences. For vowels, the results contradict those of previous studies that suggest universal emotional responses, depending on the culture. This disparity is likely because China uses a tonal language, unlike Korea. While Korean has a dialect with a different pitch from that of the standard language, it is not a tone language that conveys variations in meaning through pitch differences. By contrast, China's tonal language facilitates meaning changes through changes in tone. Therefore, Korean and Chinese listeners may experience differences in arousal and valence patterns when hearing the same sound. This finding is supported by the results of a study comparing the vowels /a/, /u/, and /i/ in Chinese with tone and English without tone, highlighting differences in sound symbolism based on the presence or absence of lexical tones (Chang et al., 2021).

We aimed to observe the differences between wakefulness and emotional outcomes. Clear and distinct differences were apparent in wakefulness, whereas in emotion, only the interaction between nationality and consonants proved significant. To elucidate, the most substantial difference between Korean and Chinese syllables, apart from phonemic constraints, lies in the pronunciation elements through the presence of sound. Korean allows for seven syllable-final consonants: ᄇ /p/, ᄃ /t/, ᄀ /k/, ᄆ /m/, ᄂ /n/, ᄋ/η, and ᄅ /l. By contrast, in Chinese, syllable-final consonants are restricted to two: /-n/ and /η/. The disparity in syllable-consonant combination constraints is the primary cause of phonological variation between Korean and Chinese.

The importance lies not only in the difference in the phoneme itself but also in the interaction effect between nationality and voice gender. Differences in the arousal and valence experienced in response to Hangul phonemes varied by nationality depending on whether the voice was female or male. In particular, Chinese women were found to experience negative emotions even when the voiced sound was presented with a male voice, although voiced consonant is an articulation method that results in less arousal than lenis and aspirated consonants. Although the study was conducted among Chinese participants living in Korea rather than in China, cultural values do not change easily (Hofstede, 1984, 1998), which may be attributed to cultural differences based on gender roles. According to a cultural-level study, China has a wider gender power gap than Korea; China highly values the image of masculinity (Moon and Woo, 2019), and Chinese women feel that they are not free to express themselves and are restricted in their opportunities to demonstrate their abilities because of men (Sun, 2022). Consequently, Chinese women are less dependent on men and experience a sense of competition with them. Therefore, compared with Korean participants, Chinese participants experienced more negative emotions toward a male voice than a female voice. This trend can be further clarified through research on cultural differences in gender-related issues.

In recent years, the generation and interpretation of literary works and various forms of literature through AI have highlighted the increasing importance of studying people's emotional responses. Specifically, the examination of the ability of AI to convey emotions or impart specific emotions is important. Currently, by utilizing natural language processing and machine learning techniques, research studies investigate how emotions are expressed and recognized in literary works, as well as how the tone and expression AI employs when narrating stories evoke emotional responses in listeners (Spezialetti et al., 2020; Lettieri et al., 2023). This study aimed to determine the emotional responses elicited in listeners by the tones and expression styles used by AI when delivering verbal expressions. Furthermore, in an era marked by an active international approach to media use, this study is significant in exploring the specific differences in emotional responses to voices in Chinese and Korean, languages characterized by distinct intonations, and cultural and political expressions despite their geographical proximity.

## Limitations

This study has several limitations. First, although the study focused on AI, various AI voices were not used for the investigation. Various AI speakers have been released in both Korea and China, and people exhibit different preferences. Therefore, the sound stimuli used in this study are not perfectly consistent with the degree of arousal and valence experienced with the currently available voices of the AI speakers. For marketing applications, further research using the voices of various AI speakers is needed. In addition, some AI speakers now allow customers to purchase celebrity voices that they like directly, in which case, the results of this study would be challenging to apply, even for male voices.

Second, the study did not include comparisons with other cultures. Both Korea and China are cultural regions in Northeast Asia, but subtle differences may occur across various languages and cultures. Although Korea and China are in the same region, the fact that differences were found according to voice gender indicates that differences based on voice gender may occur in other cultures and may be relatively larger. In the future, multicultural studies should be conducted to compare a wide range of languages and cultures.

Third, this study was conducted with female participants. Women tend to respond more emotionally than men, and only women were recruited based on the existing claim that sound symbolism does not differ significantly by gender. However, cultural differences may interact with gender.

Finally, this study used only three vowels (/a/, /u/, and /i/) and 14 consonants; however, fortis consonants were not used. In Chinese, intonation plays a significant role alongside pronunciation in conveying the meanings of individual words. Hence, future research should meticulously examine variations in intonation within words of identical pronunciation to ascertain their emotional impact conveyed through speech. In addition to articulation, intonation is also used in China. Therefore, a more detailed classification is required to reflect the actual situation.

Despite these limitations, this study is significant in demonstrating that arousal and valence may differ in articulation types and vowels depending on cultural differences, and that voice gender can also affect perceived emotions. This principle supports sound symbolism and has practical implications for voice gender and branding in AI applications.

## Data availability statement

All data can be made available upon request to the corresponding author.

## Ethics statement

The studies involving humans were approved by the Institutional Review Board of Chung-Ang University (IRB NO. 1041078-20230210-HR-032). The studies were conducted in accordance with the local legislation and institutional requirements. The participants provided their written informed consent to participate in this study.

## Author contributions

M-SL: Conceptualization, Data curation, Formal analysis, Investigation, Methodology, Visualization, Writing— original draft. G-EL: Conceptualization, Data curation, Formal analysis, Project administration, Validation, Writing—original draft, Writing—review & editing, Supervision. SL: Conceptualization, Funding acquisition, Project administration, Validation, Writing—review & editing, Supervision. J-HL: Conceptualization, Project administration, Validation, Writing—review & editing, Supervision.
